# Understanding Meditation Based on the Subjective Experience and Traditional Goal: Implications for Current Meditation Research

**DOI:** 10.3389/fpsyg.2019.01827

**Published:** 2019-08-21

**Authors:** J. Shashi Kiran Reddy, Sisir Roy

**Affiliations:** Consciousness Studies Programme, National Institute of Advanced Studies (NIAS), Indian Institute of Science (IISc) Campus, Bangalore, India

**Keywords:** meditation, meditative state, Turiya, subjective experience, pure consciousness, states of consciousness, neural oscillations

## Abstract

Owing to its benefits on various cognitive aspects, one’s emotions, and well-being, meditation has drawn interest from several researchers and common public alike. We have different meditation practices associated with many cultures and traditions across the globe. Current literature suggests significant changes in the neural activity among the different practices of meditation, as each of these practices contributes to distinct physiological and psychological effects. Although this is the case, we want to find out if there is an underlying commonality among all these different practices. Thus, we ask the following questions related to different practices of meditation, the traditional goal of meditation and its significance—what is the central purpose of meditation? Do traditions define the final goal of all the practices of meditation? Are the purpose and goal of these practices different or is there a common goal to be attained through all these distinct practices? Embracing the traditional perspective, through this paper, we want to emphasize that, although these techniques and practices may appear different on the periphery, eventually, they seem to subject one to the same experience at the end, a natural meditative state (discussed in various spiritual traditions as the goal of meditation). In view of future studies on different meditation practices and also those exploring this subjective state, we offer some interesting ideas based on the traditional insights into meditation. In this context, we would also like to make a few comments on the way contemporary researchers view different practices of meditation.

## Introduction

In the last two decades, interest in meditation has grown among the common public as a practice for well-being. Thus, it raised a curiosity in the scientific community to study the neural correlates of meditation to assess various such claims. In this connection, we have many studies aimed at tracing the neural patterns underpinning the effects caused by meditation practices (Ospina et al., 2007; Tang et al., 2015; Tomasino and Fabbro, 2015; Braboszcz et al., 2017). Now, there is preliminary evidence supporting the beneficial effects of meditation on various cognitive aspects, one’s emotions, and well-being (Braboszcz et al., 2010; Schmidt, 2014; Schmidt and Walach, 2014; Black et al., 2015; Simon and Engstrom, 2015; Tang et al., 2015; Luberto et al., 2017). In these studies, they generally use the term meditation to identify a set of cognitive techniques and contemplative practices. Accordingly, we have various meditation practices associated with numerous cultures and traditions across the globe (Shear, 2006; Manocha, 2011; Awasthi, 2013; Nash and Newberg, 2013; Schmidt, 2014; Tomasino et al., 2014; Bærentsen, 2015). Since each type of practice leads to different somatic and psychological effects, these practices influence different regions of the brain. For instance, many recent findings indicate that the practice of meditation can result in both short-term and long-term structural and functional brain changes, but these effects are specific to each type of meditation (Lutz et al., 2008; Braboszcz et al., 2010; Fell et al., 2010; Vago and Silbersweig, 2012; Fox et al., 2014, 2016; Lippelt et al., 2014; Tomasino et al., 2014; Tang et al., 2015).

To have an in-depth understanding and consistent overview of which type of meditation results in a specific effect, it is important to review the research studies on each of these practices. Of note is one such recent attempt made in Lee et al. (2018), which presents a systematic review of the literature with the aim of studying the neural oscillations underlying meditation. Here, the authors reviewed the following four most commonly studied meditation practices: focused attention (FA), open-monitoring (OM), as well as transcendental meditation (TM), and loving-kindness (LK) meditation. The authors report that current literature suggests significant changes in the neural oscillatory activity (of cortical and subcortical areas) among these four meditation practices. Their findings show that meditation is characterized by the global increases in oscillatory activity among practitioners compared to meditation-naïve subjects, with overall changes being positively correlated to the length of training/practice.

While they report similar gamma activity in the frontal areas during the practice of FA, OM, TM, and LK meditation, changes are variable in parietal and occipital areas. Overall, different meditation practices are characterized by changes in theta, alpha, and gamma activity in the anterior and posterior regions of the brain. For instance, increased alpha coherence and activity is reported in the frontal and parietal regions during FA, OM, and TM (Travis, 2001; Cahn et al., 2013; Travis and Parim, 2017), but the predominant oscillation in TM is the frontal alpha in contrast to theta in FA and OM. With the advent of imaging technologies such as functional magnetic resonance imaging (fMRI), now it has also become easy to study the functional connectivity between different regions of the brain during any particular task/activity such as meditation. The fMRI studies on meditation practices also support distinct functional connectivity associated with different meditation practices (Brewer et al., 2011; Fox et al., 2014, 2016). Considering the number of available practices of meditation with their physiological and psychological influences, the research literature on meditation is exhaustive; thus, we recommend reading such work elsewhere (Takahashi et al., 2005; Lutz et al., 2008; Fell et al., 2010; Vago and Silbersweig, 2012; Lippelt et al., 2014; Tomasino and Fabbro, 2015; Travis and Parim, 2017; Chau et al., 2018).

As suggested in Lee et al. (2018), in view of the current state of research, more rigorous studies are needed to elucidate the nuanced imaging and electrophysiological changes that occur with each type of meditation. Such studies will help us in understanding how these practices differ in their effects, following which, one can develop different meditation-based interventions for any specific task. For example, now it is known that several neuropsychiatric disorders are characterized by altered neural oscillations (Simon and Engstrom, 2015; Lee et al., 2018), so studying the oscillatory patterns associated with different practices of meditation (and their potential influence) could help in devising meditation-based clinical interventions for various medical disorders. Lately, such interventions are developed to cultivate prosocial behaviors as well (Reddy and Roy, 2019).

However, is that all a meditation is about? Here, we summarize some interesting questions about meditation and its traditional significance to further motivate the discussion in the present article—what is the central purpose of meditation? Where will these different techniques of meditation lead an advanced meditator with long-term practice? Do traditions define the final goal of all these practices of meditation? Are the purpose and goal of these practices different or is there a common goal to be attained through all these distinct practices? While searching for answers to these questions, we reviewed the traditional literature associated with various cultures that introduced these ancient practices to the modern world (Woods, 1927/2003; Rukmani, 2001; Lama et al., 2003; Shear, 2006; Manocha, 2011). From this perspective, we offer a novel framework and interpretation to look at different practices of meditation.

## On the Traditional and Contemporary Views of Meditation

In this section, we explore if the contemporary view of meditation agrees with the traditional view, and if not so, how does it differ? After analyzing the contemporary and traditional views of meditation (as per the available literature and research), we summarize the following observations:

1. Many contemporary researchers consider or define meditation as a set of cognitive techniques involving attention, concentration, and observational task. Even though this justifies the reason all such techniques and practices fall under one umbrella term—meditation, there is a discrepancy regarding meditation practices emanating from different traditions, as they do not satisfy a common definitional criterion. Thus, there are taxonomical, definitional, and methodological issues when we attempt to study these practices empirically. A detailed discussion on such issues and how to overcome some of them while conducting different studies on meditation can be found elsewhere (Rao, 2011; Awasthi, 2013; Nash and Newberg, 2013; Kreplin et al., 2018).
2. In addition, meditators following a particular tradition would not agree that their meditation practice is a simple cognitive task or a mere cognitive technique. Traditions often define a specific goal for practicing meditation and also involve details about the different levels and stages of meditation one will come across as one progresses toward the goal (Lama et al., 2003; Shear, 2006).
3. Much effort in current meditation research is dedicated to understanding the neural correlates of meditation and developing meditation-based interventions. Very few studies have been done in understanding different stages, levels, and goal of meditation (which is extensively discussed in the traditional spiritual literature; Woods, 1927/2003; Rukmani, 2001; Lama et al., 2003; Shear, 2006; Manocha, 2011).
4. In most of the scientific studies on meditation, the purpose of meditation is either ill-defined or overlooked. Meditation practice may either have short-term or long-term goals—either to understand the workings of one’s own mind and realize one’s true nature of the self (as dictated by the traditions) or other goals (which include positive clinical implications, well-being, etc.,).
5. It will be interesting to see where the current research on meditation stands in understanding the various stages and levels of meditation, besides understanding the goal of meditation (as indicated by different traditions). In relation to studying the goal of meditation, another aspect that should be considered in meditation research is the subjective experience associated with different practices and with the end goal of meditation.
6. We consider that one may understand meditation better if we view these practices as discussed in the spiritual literature. Therefore, there is a need to understand meditation based on the traditional perspective, which may offer novel insights to analyze current meditation research.

Accompanying some of the above observations in the present article, we explore if there is a possible commonality underlying all these diverse practices of meditation. In this context, the current work is uniquely different from other studies in this area. Many studies have previously attempted to look for such commonality, but they base their findings on the objective measurements alone, leaving the subjective aspect of the meditative experience (Braboszcz et al., 2010, 2017; Tomasino et al., 2014; Lee et al., 2018). As stated above, one cannot consider meditation as a mere physical exercise or technique alone; the subjective experience of a meditator has to be considered as a crucial aspect in meditation research. At the onset of practice, the experience seems to vary based on the meditation technique. For instance, initially FA and OM meditation will cause different subjective experiences because of the usage of different cognitive modalities; one requires the focused attention on the object of meditation (which varies from tradition to tradition) and the other involves the objectless and open awareness.

In the present context, considering the number of practices and techniques we have across different traditions and cultures, it would be relevant to revisit the questions we asked at the beginning of the discussion—are these techniques developed for different functional purposes? Do all these techniques result in different meditative states or are these devised to reach the same state of subjective experience? A meditative state can be viewed as a final subjective state of experience that a particular technique would eventually result in, in the end (Rao, 2011; Reddy and Roy, 2018a). To answer these questions, at first, we would need to study the intention and final goal set by different traditions and cultures that devised these techniques (Woods, 1927/2003; Rukmani, 2001; Lama et al., 2003; Shear, 2006; Manocha, 2011; Baars, 2013; Bærentsen, 2015; Reddy and Roy, 2018a). Since these traditions introduced meditation techniques to us, it is important to refer to them to know the essentials involved in the practice.

Some contemporary versions of different meditation practices differ much from their ancient practices both in the way they are practiced and in their final goals and intentions (Rao, 2011; Awasthi, 2013; Monteiro et al., 2015; Reddy and Roy, 2019). However, in general, this is not the case. The traditional practices look different in the way they are practiced, although these are motivated by a common intention and goal. A deeper analysis of various meditation practices emanated from distinct traditions reveals that, through all these practices, they intend to attain the same goal (or meditative state). We found that the purpose (or end goal) of meditation is to experience a unique subjective state. It is to cultivate an inner state of equanimity, and eventually, attain Buddhahood or the state of Samadhi (in simple terms, an experience close to a thoughtless state: Woods, 1927/2003; Rukmani, 2001; Lama et al., 2003; Shear, 2006; Manocha, 2011; Rao, 2011; Bærentsen, 2015). In a different context, we recently reported this goal as a natural meditative state (Reddy and Roy, 2018a), and the reason why we address this state as a natural meditative state will be made clear in the next section.

Hence, various meditation techniques were devised with a primary goal of experiencing this subjective state in one’s lifetime. Though different practices are associated with varying experiences at the onset, these practices will eventually lead an individual to the same inner state of experience at the end. Thus, techniques and practices may appear different on the periphery, but they are all aimed at a common goal. This is where the outer diversity in practices collapses to an inner conscious experience. Thus, we find it crucial to analyze the current scientific studies by taking this subjective state as the basis.

From the traditional perspective, meditation as a practice comprises different onset techniques, various stages of evolution (as one progresses), and the final goal (Woods, 1927/2003; Rukmani, 2001; Lama et al., 2003; Shear, 2006; Reddy and Roy, 2018a). Hence, meditation essentially involves practicing a technique through which a meditative state is experienced (as the end goal). Various experiences resulting from the practice of meditation are relative markers for evaluating how deep a person has gone in the experience of meditation. For example, various traditional texts discuss the experiential states of a person at a particular level/stage of evolution. The practice may involves either 4 to10 stages or different (more or less stages) based on the tradition it represents. It is interesting to see that, although there is a difference in several stages of meditation that are associated with varying experiences, the end goal seems to be the same. Recently, an attempt to study a stage-wise evolution of meditation practices has been made in the following studies: Telles et al. (2015), Kok and Singer (2017), and Deepeshwar et al. (2019). Here, the authors report how the stage-wise neural activity varies as one progresses in the practice of meditation. In addition, one should also note that, as considered in most of the scientific studies of meditation, in spiritual traditions, a person’s experience (in a particular meditation technique) is evaluated not just based on the number of hours and years of practice but based on the level of inner subjective experience. They base such an evaluation of the pre-defined goal and various stages one would come across from the time they begin the practice until one reaches the final state (as discussed above). Hence, we may need a different basis to compare subjects in meditation research, and in this context, the subjective experience of a meditator appears to be a crucial aspect to take into account. Thus, in the process of understanding meditation in its extent, we need to study the different stages of meditation from the onset of practice until the goal is attained. This will also help us in knowing various factors that influence one’s progress in meditation.

Besides several methodological and taxonomical issues noted by some researchers (Rao, 2011; Awasthi, 2013; Nash and Newberg, 2013; Schmidt, 2014; Reddy and Roy, 2018a), there seem to exist various biases while conducting experimental studies on meditation. In Kreplin et al. (2018), authors cautioned that sometimes methodological frailties and various biases involved in the study such as those introduced by beliefs and expectations on the power of meditation, type of control group selection, and meditation instructor could also influence the outcome and result. Hence, there is a need for traditional insights into meditation to develop a novel framework for analyzing current meditation research. In this regard, it will be interesting to study not only different practices of meditation objectively but also the associated and relative states of subjective experience.

We begin the following section with the discussion about the subjective state (which is seen as the goal of meditation)—a natural meditative state, and then discuss some studies that attempted to capture this subjective experience using scientific modalities.

## The Traditional Goal of Meditation: A Natural Meditative State

To understand meditation in its extent, it is essential to explore the aspects of how, why, and what of meditation. In previous sections, we discussed to some extent the aspects of what and how of meditation, and here, we focus on understanding “why” meditation. This essentially deals with the purpose of why one should practice meditation or why these practices are developed in the first place. Thus, in addressing the context, the goal of meditation and the experiential state associated with it (as indicated by different traditions) have to be studied.

Ancient traditions often describe this subjective experience as a state of being that transcends the normal states of experience (Rukmani, 2001; Nikhilananda, 2006; Shear, 2006; Manocha, 2011). The following lines taken from the Mandukya Upanishad describes the subjective state of what one believes to be the goal of meditation:

…not which is conscious of the inner (subjective world), nor that which is conscious of (objective) world, nor that which is conscious of both… It is unperceived (by any sense organs), incomprehensible (to the mind), unrelated (to any object), un-inferable, unthinkable, and indescribable. It is essentially of the nature of consciousness… (Nikhilananda, 2006).

One can find references to such subjective experiences in almost all the traditions that introduced meditation practices to the world (Travis and Pearson, 2000; Shear, 2006). In the Vedantic literature, it is known as Turiya (Ramamurthi, 1995; Nikhilananda, 2006). It is also referred to as Samadhi in the Yogic culture (Hinduism), Nirvana/Buddhahood in Buddhism, and Satori in Zen (Rukmani, 2001; Manocha, 2011; Rao, 2011). The state of awareness associated with this experience is unique from other experiences (including many spiritual and religious experiences), as it is supposed to have no mental content of experience (Woods, 1927/2003; Travis and Pearson, 2000; Rukmani, 2001; Shear, 2006; Rao, 2011; Baars, 2013). For this reason, such a state is also referred to as consciousness without content or content-free consciousness. Some contemporary authors even discussed this state as pure consciousness, silent consciousness, thoughtless state, non-dual experience, mental silence, and the fourth state of consciousness. (Shear and Jevning, 1999; Travis and Pearson, 2000; Manocha, 2011; Josipovic, 2013, 2019; Hernandez et al., 2015; Turjman, 2018).

As per the ancient traditions, a non-dual state of awareness signifies this subjective state. In general, when we consider a normal experience, there exist two main components: one is the experiencer (the subject) and the other is the object of experience. In the context of meditation, a non-dual experience can be reported from two different perspectives (based on the type of practice): one is the subject-referral and the other is the object-referral experience. In normal conditions, the subject is aware of the object and can differentiate between oneself and the other (or object). However, in the object-referral non-dual experience, the sense of identity (which is identified in the traditional literature as ego) is lost, and hence the meditator becomes one with the experienced object and claims to register no thought in connection to the object. The recognition of the object happens in one’s consciousness only when one can differentiate him/herself from the other; the moment the sense of identity is lost, the duality associated with any subjective experience is lost. On the other hand, the subject-referral non-dual experience could happen when the practice involves the attention or awareness of the inner self of the subject (Travis and Shear, 2010). Here, the subject himself/herself becomes the object of his/her awareness. As one progresses and practice becomes much deeper, this manifests in a subject referral non-dual experience. This state can be characterized by the non-presence of discursive thinking and bounded objects of consciousness. Lately, a few attempted to study the neural correlates associated with this kind of non-dual awareness (Travis and Shear, 2010; Josipovic, 2013, 2019; Berman and Stevens, 2015; Turjman, 2018). The findings from these studies are discussed later in this section. Considering the current state of research on meditation, in the future, we need more such studies in the context of meditation to have an in-depth understanding of this subjective state.

Another interesting point to note is that, although the ancients devised various techniques like meditation to get a glimpse of this experience and stabilize oneself in that state, they also claim that this is not a new state to be attained. This is because, according to them, one is already functioning in this state of awareness and it underlies all other normal states of consciousness like waking, dreaming (REM), and deep (non-REM) sleep states (Shear and Jevning, 1999; Sharma, 2004; Nikhilananda, 2006; Cvetkovic and Cosic, 2011; Travis, 2011; Parker, 2019). They also indicate that this state acts as a substratum on which other states of consciousness happen and are recognized. Grounded on this view, this state is also referred to as a natural meditative state (Reddy and Roy, 2018a,b). Travis, in his studies, while exploring the neural correlates of pure consciousness, adopted this perspective (Travis and Pearson, 2000; Travis, 2011). Here, the authors looked for the presence of the state of awareness that underlies all three states of consciousness (waking, dreaming, and deep sleep states). Their findings suggest that pure consciousness is characterized by unique phenomenological and physiological correlates. The presence of autonomic orienting at the onset of breath changes, apneustic breathing, and increased frequency of peak EEG power are some physiological features associated with pure consciousness. To model such a state, they developed the “Junction Point Model (JPM)” to show how pure consciousness is unified with all other states of consciousness. These findings are mainly used for comparing and evaluating the experiences reported by the advanced meditators practicing transcendental meditation (TM).

As considered by the ancients, if this subjective state is not new to our experience, then one may ask, why do we need practices like meditation in the first place? In answering this, ancients claim that one becomes aware of this state (or realize it) only in a particular functional state that goes beyond our present understanding of consciousness. This state of realization can happen to a few individuals spontaneously and naturally, but to others, only through the practice of various techniques and methods like meditation. In some recent studies (Schmidt and Walach, 2014; Tomasino et al., 2014; Tang et al., 2015; Lee et al., 2018), since the main purpose is to explore the neural oscillations underlying meditation, we feel that it is vital to bring in the discussion about this state, which is the end goal of all such practices.

As we mentioned, studying the neural oscillations associated with this state has been attempted previously by Travis (Travis and Pearson, 2000; Travis, 2011) and also by others (Josipovic, 2013; Berman and Stevens, 2015; Turjman, 2018). Recently, this has also been explored as a state of mental silence in the context of Sahaja Yoga meditation (Hernandez et al., 2015; Hernández et al., 2018). Citing different spiritual scriptures, though all these studies claim to explore and investigate the same subjective state, there are some inconsistencies among the findings. For example, based on the phenomenal or a subjective description of the experience associated with these practices, it may appear that all these studies are reporting the same experience, but they all report different quantitative/physiological findings.

For hundreds of years, there has been a debate among philosophers and rational thinkers if it is possible to understand first-person experiences (subjective accounts) in third-person (objectively). Even if it is possible to some degree, we do not have a way to know to what extent such a translation is valid. When a normal experience itself seems to be more complex, not to think of a subjective state that ancients claim to go beyond and transcend such normal experiences. In this regard, one cannot truly evaluate if those findings in the above-mentioned studies truly indicate Turiya or pure consciousness state. In view of future studies on different meditation practices and also those exploring this subjective state, in the following section, we offer some interesting ideas based on the traditional insights of meditation.

### A Possible Framework Based on the Traditional Insights

When we refer to the different traditional literature on meditation, we will find that these traditions discussed a subjective state, which is the goal of meditation, as the baseline underlying other states of consciousness that form the core of our subjective experience of life (Ramamurthi, 1995; Travis and Pearson, 2000; Travis, 2011: Reddy and Roy, 2018a; Turjman, 2018). Thus, considering this state as a baseline (for the purpose of evaluation), we recently proposed a noise-free model of meditation (Reddy and Roy, 2018a,b). Here, we think of different meditation techniques as tools to reduce the mental noise accumulated by an individual in his/her lifetime. This view is developed according to the traditional repository on yoga and meditation: Patanjali Yoga Sutras (Woods, 1927/2003; Rukmani, 2001). Sage Patanjali, in this text, indicated that mental noise may exist in various forms as different “samskaras” (subtle mental impressions or psychological imprints). Following this, we propounded that different meditation techniques essentially reduce types of noises by dealing with different cognitive modalities but finally lead to the same state of experience, a noise-free state or a natural meditative state (Reddy and Roy, 2018a,b). As discussed in previous sections, experiencing or realizing the presence of this natural meditative state is often referred to as the goal or purpose of meditation. Since this goal can be subjectively considered as the noise-free state, now, it becomes clear as to why some authors refer to this as a state of mental silence. The above perspective of meditation not only supports the traditional understanding but also establishes a universal baseline for comparing subjects (which is one of the major issues faced in the current meditation research). In the future, we would need to advance this model further to develop a novel methodology to conduct empirical studies.

When we try to analyze meditation from a different perspective, it is also important to note that some meditation techniques are not complete practices by themselves. They are just techniques to attain a particular functional state of experience. Either each practice involves different stages of progress or they themselves make part of a level/stage-wise arrangement leading to the goal. In the latter instance, after learning one technique of meditation, one may need a different technique to progress further toward the goal set by the respective tradition. Thus, as usually done in most meditation studies, it is important not to consider each technique as an independent practice by itself, though it is always helpful to know the details of each step leading to a goal. If we consider one’s goal (as a meditator) is to be aware of the natural meditative state in each one of us, all these different techniques may have been devised stepwise to transcend a particular cognitive function to bring in the realization. With some limitations, since we now have a good amount of data on each different type of meditation (at least, those considered in Lee et al., 2018), it is important to integrate some practices and see if all these techniques in a particular combination result in a final meditative state. As indicated by Sage Patanjali, if all these techniques are considered as tools to reduce or subside the noises developed as a consequence of different mental content, then what remains when we remove these noises is one’s natural meditative state (which is free from any mental content). It will be very interesting to see if all these practices lead to this base state.

From all the above discussions, it seems that the natural state is common not only to any practice of meditation but also to all other states of consciousness. Although current research on meditation helped us in developing various meditation-based interventions and target-specific techniques to tackle certain health conditions, one should not forget the essential purpose as to why ancients developed these techniques. It will be interesting to study the subjective state associated with the goal of meditation as attempted by some researchers (Ramamurthi, 1995; Travis and Pearson, 2000; Rao, 2011; Travis, 2011; Baars, 2013; Josipovic, 2013; Berman and Stevens, 2015; Turjman, 2018), but with better methodology and study design to overcome the inconsistencies that exist with current research. In light of the extensive interest in understanding consciousness and in developing and devising various clinical interventions, it is crucial to look for the possible existence of such a subjective state, which is known to be underlying the other three states of consciousness. Some researchers have already indicated the possible existence of such a state as the basis for any subjective experience (Guay and Schanker, 2018; Sullivan, 2018; Fingelkurts and Fingelkurts, 2019). Here, they have explored it in the context of defining consciousness in the conditions of minimal awareness. Such studies will help us not only in understanding the nature of our own consciousness but may also open up new ways to devise various clinical methods for health conditions associated with different altered states.

Considering the possible research challenges, it may not be that simple as it appears to study the subjective state associated with the goal of meditation. In this scenario, it would be beneficial to study the subjective experience associated with different practices of meditation and tracking the stage/level-wise experiences. Some studies have already been conducted (independently by different groups) to assess the levels and various kinds of subjective experiences associated with different practices. One group correlates these experiences with the neurophysiological and neuropsychological data (Telles et al., 2015; Kok and Singer, 2017; Singer and Engert, 2018; Deepeshwar et al., 2019), whereas others study and discuss the phenomenological and subjective aspects (Shear and Jevning, 1999; Varela and Shear, 1999; Shear, 2006, 2013). Since meditation as a practice stems from ancient spiritual cultures, we feel that there is a need to bridge empirical investigations with philosophical and phenomenological approaches as discussed in the traditional literature. Thus, one should not only design a study, but also analyze meditation according to the traditional insights provided in ancient literature in order to avoid various methodological and taxonomical issues. Thus, empirical studies on meditation should take into consideration various philosophical and phenomenological aspects while designing a study.

In addition, neuroplastic changes associated with the practice of meditation can help in tracing different levels/stages as well as one’s progress (Davidson, 2005; Chau et al., 2018). This is because the plastic changes induced by these practices are neither immediate nor spontaneous. They can be continuously and progressively mapped and monitored in long-term practitioners. Thus, neuroplastic changes allied with any practice of meditation can give us some indication on different levels/stages of meditation. Once we study the kind of experiences involved in distinct practices and their stage-wise evolvement, we may have some insights into how to model and study the end experience—a natural meditative state.

As mentioned earlier in this article, many researchers previously specified that current meditation research suffers from various issues caused either because of adopted methodology and study design (as they are not in accordance with traditional practice) or because of other possible biases. To avoid some of those issues in future scientific studies on meditation, we suggest that empirical investigations involving experimental studies on meditation should also account for qualitative/psychological assessments. It will be interesting to conduct the simultaneous assessment of both the psychological and physiological data and later correlate them. Most of the studies focus on investigating either of the data, but not both in a single study. Such simultaneous assessment may or may not offer much information in relation to other specialized cognitive tasks, but in the case of meditation, as dictated by tradition, it looks crucial to assess experience alongside the physiological investigations.

One reason why it is so important to evaluate the experience of the subjects alongside the physiological monitoring is that there may arise situations where the final outcomes and results of the study are influenced by different biases, expectations, and prejudices (Kreplin et al., 2018). Knowing the influential role these factors could play in measurements, it will be difficult to comment to what extent meditation as a practice can result in the desired output. In order to overcome such issues and truly understand the potential of meditation, we need to develop different questionnaires to assess subjects (for various aspects) and use them alongside the tests that monitor physiological data. This gives the estimate of the number of different biases that could interfere with the final outcome. For instance, these questionnaires should give basic information such as what do the subjects know about meditation? Why do they choose a particular form of meditation? What appeals to them more in the following practice? What do they expect from the meditation practice? And finally, whether they have reached their expected level or not after the practice session, etc., This information has to be correlated with the observed experimental results, to find out how meditation (as a practice by itself without other influential factors) can impact an individual.

## Conclusion

Many studies have previously initiated the discussion on the purpose of meditation by attempting to study objectively the neural oscillations underlying different meditation practices. Based on the traditional perspective on meditation, to this very discussion, we bring in the concept of a natural meditative state as the goal of meditation. Outwardly, though we seem to have different practices of meditation (emanated from distinct traditions), a deeper analysis of such practices reveal that their goal is to attain the same state of meditative experience. All these techniques are aimed at reaching a particular state of consciousness, which is a natural meditative state. Although few attempted to study this state using scientific modalities, currently we do not have a consistent understanding regarding the possible existence of such a state. Through the present paper, we wish to initiate the discussion about this subjective state and call for more studies in the context of meditation research. Such studies will have implications for our fundamental understanding and may offer novel insights into the nature of consciousness. In addition, to avoid various issues, we propose that experimental studies on meditation should also gather general information and monitor psychological data simultaneously by using different questionnaires. Later, both these data should be correlated to see if experimental data are influenced by any potential factors such as expectations, prejudices, other biases, etc., Such a study design offers a possibility to study and understand meditation in its true extent.

## Author Contributions

All authors listed have made a substantial, direct and intellectual contribution to the work and approved it for publication.

### Conflict of Interest Statement

The authors declare that the research was conducted in the absence of any commercial or financial relationships that could be construed as a potential conflict of interest.
